# Crystal structure of 3-[(2-acetamido­phen­yl)imino]­butan-2-one

**DOI:** 10.1107/S2056989018000749

**Published:** 2018-01-19

**Authors:** Feng Zhai, Joseph B. Solomon, Alexander S. Filatov, Richard F. Jordan

**Affiliations:** a Department of Chemistry, The University of Chicago, 5735 South Ellis Ave, Chicago, Il 60637, USA

**Keywords:** crystal structure, imino­ketone, hydrogen bonding

## Abstract

In the title compound, 3-[(2-acetamido­phen­yl)imino]­butan-2-one, C_12_H_14_N_2_O_2_, the imine C=N bond is essentially coplanar with the ketone C=O bond in an *s-trans* conformation. The benzene ring is twisted away from the plane of the C=N bond by 53.03 (14)°. The acetamido unit is essentially coplanar with the benzene ring. In the crystal, mol­ecules are connected into chains along the *c* axis through C—H⋯O hydrogen bonds, with two adjacent chains being hinged by C—H⋯O hydrogen bonds.

## Abstract 2

In the title compound, the imine C=N bond is essentially coplanar with the ketone C=O bond in an *s-trans* conformation. In the crystal, mol­ecules are connected into chains along the *c* axis through C—H⋯O hydrogen bonds, with two adjacent chains hinged by C—H⋯O hydrogen bonds.

## Chemical context   

α-(Aryl­imino)­ketone compounds, resulting from condensation between α-diketones and anilines in a 1:1 fashion, are useful bidentate ligands in transition metal coordination chemistry (Binotti *et al.*, 2004 ▸) and important synthetic inter­mediates toward α-di­imines (Schmid *et al.*, 2002 ▸) and imine-based multidentate ligands (Schmiege *et al.*, 2007 ▸). X-ray structural studies of α-(aryl­imino)­ketones have primarily focused on those derived from aromatic diketones such as acenaphthene­quinone (Kovach *et al.*, 2011 ▸), benzil (Kovach *et al.*, 2014 ▸; Güner *et al.*, 2000 ▸), and phenanthrene­quinone (Farrell *et al.*, 2017 ▸). In contrast, structural reports on α-(aryl­imino)­ketone compounds derived from aliphatic α-diketones are rare (Azoulay *et al.*, 2009 ▸).

Our group is inter­ested in *N,N*-diaryl α-di­imine ligands that contain hydrogen-bonding units for transition-metal-catalyzed copolymerization of polar vinyl monomers with ethyl­ene (Zhai & Jordan, 2014 ▸; Zhai *et al.*, 2017 ▸). We obtained the title compound during the attempted synthesis of an α-di­imine compound containing an *ortho*-acetamido group and report its crystal structure in the present work.
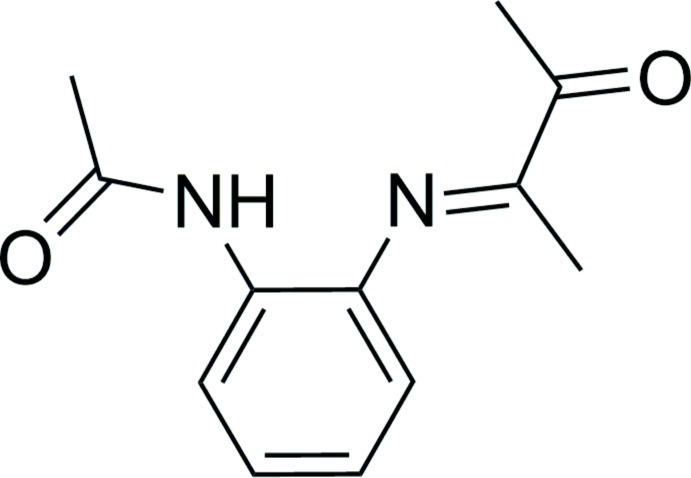


## Structural commentary   

**Figure 1:**
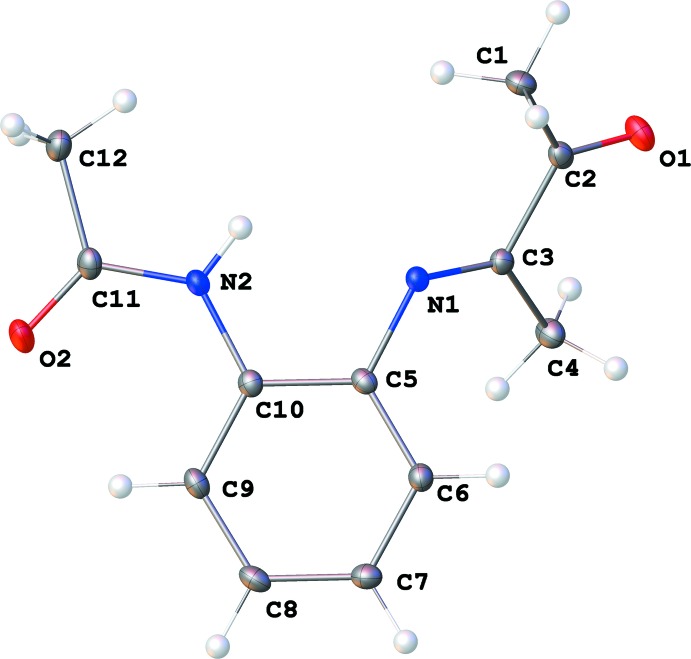
The mol­ecular structure. Displacement ellipsoids are shown at the 50% probability level.

The mol­ecular structure of the title compound is shown in Fig. 1 ▸. The aryl­imine unit exhibits an *E* conformation. The ketone carbonyl group (C2–O1) and the imine C=N group (C3–N1) are almost coplanar [torsion angle O1—C2—C3—N1 −177.87 (10) °] and *trans* with respect to the C2—C3 bond. The imine plane is twisted from the plane of the aryl ring (C5–C10) by a dihedral angle of 53.03 (14)° [defined by atoms C3/N1/C5/C6]. The acetamido group is essentially coplanar with the aryl ring [torsion angle C11—N2—C10—C9, −0.14 (18)°]. The mol­ecular structure of **I** also features intra­molecular C9—H9⋯O2 hydrogen bond (Table 1 ▸). This bond, in combination with conjugation between the amide group and the aryl ring, is likely responsible for the coplanarity between the acetamido and the aryl groups.

## Supra­molecular features   

**Figure 2:**
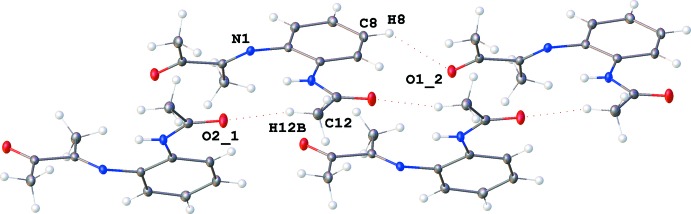
Chains running along the *c-*axis direction. [Symmetry codes: (_1) *x*, −*y* + 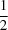, *z* + 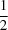; (_2) *x*, *y*, *z* − 1.]

**Figure 3:**
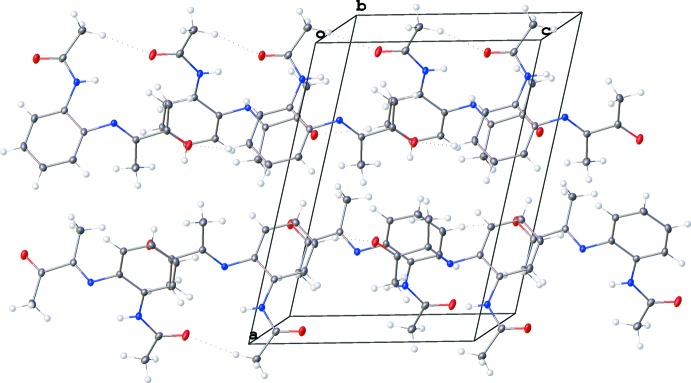
Crystal packing of the title compound.

In the crystal, C8—H8⋯O1^ii^ [symmetry code: (ii) *x*, *y*, *z* − 1 hydrogen bonds arrange the mol­ecules into chains along the *c* axis (Fig. 2 ▸, Table 2 ▸). Two chains in close proximity are linked by C12—H12*B*⋯O2^i^ hydrogen bonds [symmetry code: (i) *x*, −*y* + 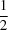, *z* + 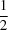]. There are no other significant contacts between the chains (Fig. 3 ▸).

## Database survey   

A search of the Cambridge Structural Database (CSD, Version 5.38, update May 2017; Groom *et al.*, 2016 ▸) indicated that no other α-(aryl­imino)­ketone compounds derived from 2,3-butane­dione have been structurally characterized. Two structurally similar α-(aryl­imino)­ketones have been reported, namely 2,4-bis­(2,6-diiso­propyl­phenyl­imino)­pentan-3-one [CCDC refcode COPLAV (Azoulay *et al.*, 2009 ▸) and its identical structure COPLAV01 (Zhang *et al.*, 2012 ▸)] and 2-(2,6-diiso­propyl­phenyl­imino)-1-phenyl­propan-1-one (IFA­DAV; Ferreira *et al.*, 2006 ▸).

## Synthesis and crystallization   

A Schlenk flask was charged with *N*-(2-amino­phen­yl)acetamide (Shirin *et al.*, 2002 ▸) (2.00 g, 13.3 mmol) and anhydrous MeOH (11 mL) under nitro­gen. The mixture was cooled to 273 K. Butane-2,3-dione (2.30 g, 26.7 mmol) and a catalytic amount of formic acid (2–3 drops) were added to the reaction mixture, and the mixture was stirred at 273 K for 1 h. The mixture was warmed to room temperature, and the volatiles were removed under vacuum. The yellow solid residue was washed three times with diethyl ether and dried under vacuum to yield the title compound (2.04 g, 70%). This material slowly degrades under air at room temperature. Storage under vacuum or nitro­gen is recommended.

^1^H NMR (500 MHz, CDCl_3_): δ 8.31 (*d*, *J* = 8.0, 1H), 7.64 (*br s*, 1H, N*H*), 7.24 (*t*, *J* = 7.5, 1H), 7.08 (*t*, *J* = 7.5, 1H), 6.78 (*d*, *J* = 8.0, 1H), 2.55 (*s*, 3H, CH_3_), 2.17 (*s*, 3H, CH_3_), 2.16 (s, 3H, CH_3_). ^13^C{^1^H NMR (126 MHz, CDCl_3_): δ 199.5, 168.0, 167.1, 136.4, 131.5, 127.7, 123.6, 120.5, 119.4, 25.1, 25.0, 14.9. Single crystals were obtained from diffusion of diethyl ether into a THF solution at room temperature under nitro­gen.

## Refinement   

Crystal data, data collection and structural refinement details are summarized in Table 2 ▸. Carbon-bound H atoms were placed in calculated positions (C—H = 0.95–0.98 Å) and were included in the refinement in the riding-model approximation, with *U*_iso_(H) set to 1.2–1.5*U*_eq_(C). The hydrogen atom attached to the N2 atom was found in a difference-Fourier map and was freely refined without any restraints.

## Supplementary Material

Crystal structure: contains datablock(s) I. DOI: 10.1107/S2056989018000749/ld2143sup1.cif

Structure factors: contains datablock(s) I. DOI: 10.1107/S2056989018000749/ld2143Isup2.hkl

Supporting information file. DOI: 10.1107/S2056989018000749/ld2143Isup3.cdx

Supporting information file. DOI: 10.1107/S2056989018000749/ld2143Isup4.cml

CCDC reference: 1553771

Additional supporting information:  crystallographic information; 3D view; checkCIF report

## Figures and Tables

**Table 1:** Hydrogen-bond geometry (Å, °)

*D*—H⋯*A*	*D*—H	H⋯*A*	*D*⋯*A*	*D*—H⋯*A*
C8—H8⋯O1^i^	0.95	2.54	3.3286 (14)	141
C9—H9⋯O2	0.95	2.24	2.8523 (15)	122
C12—H12*B*⋯O2^ii^	0.98	2.39	3.3387 (15)	164

Symmetry codes: (i) 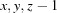; (ii) 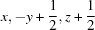.

**Table 2:** Experimental details

Crystal data	
Chemical formula	C_12_H_14_N_2_O_2_
*M*_r_	218.25
Crystal system, space group	Monoclinic, *P*2_1_/*c*
Temperature (K)	100
*a*, *b*, *c* (Å)	13.987 (3), 7.7950 (14), 10.3135 (18)
β (°)	105.556 (4)
*V* (Å^3^)	1083.3 (3)
*Z*	4
Radiation type	Mo *K*α
μ (mm^−1^)	0.09
Crystal size (mm)	0.24 × 0.18 × 0.12

Data collection	
Diffractometer	Bruker D8 Venture
Absorption correction	Multi-scan (*SADABS*; Bruker, 2015 ▸)
*T*_min_, *T*_max_	0.692, 0.746
No. of measured, independent and observed [*I* > 2σ(*I*)] reflections	25254, 2600, 2238
*R*_int_	0.045
(sin θ/λ)_max_ (Å^−1^)	0.660

Refinement	
*R*[*F* ^2^ > 2σ(*F* ^2^)], *wR*(*F* ^2^), *S*	0.038, 0.103, 1.06
No. of reflections	2600
No. of parameters	152
H-atom treatment	H atoms treated by a mixture of independent and constrained refinement
Δρ_max_, Δρ_min_ (e Å^−3^)	0.38, −0.16

Computer programs: *APEX3* and *SAINT* (Bruker, 2015 ▸), *SHELXT* (Sheldrick, 2015*a* ▸), *SHELXL2017* (Sheldrick, 2015*b* ▸), *OLEX2* (Dolomanov *et al.*, 2009 ▸) and *publCIF* (Westrip, 2010 ▸).

## supplementary crystallographic information

### Crystal data

**Table:**

C_12_H_14_N_2_O_2_	*F*(000) = 464
*M**_r_* = 218.25	*D*_x_ = 1.338 Mg m^−^^3^
Monoclinic, *P*2_1_/*c*	Mo *K*α radiation, λ = 0.71073 Å
*a* = 13.987 (3) Å	Cell parameters from 9891 reflections
*b* = 7.7950 (14) Å	θ = 3.0–28.0°
*c* = 10.3135 (18) Å	µ = 0.09 mm^−^^1^
β = 105.556 (4)°	*T* = 100 K
*V* = 1083.3 (3) Å^3^	Prism, yellow
*Z* = 4	0.24 × 0.18 × 0.12 mm

### Data collection

**Table:**

Bruker D8 Venture diffractometer	2600 independent reflections
Radiation source: micro-focus X-ray tube, INCOATEC ImuS	2238 reflections with *I* > 2σ(*I*)
Mirrors monochromator	*R*_int_ = 0.045
Detector resolution: 10.4167 pixels mm^-1^	θ_max_ = 28.0°, θ_min_ = 3.0°
ω and phi scans	*h* = −18→18
Absorption correction: multi-scan (SADABS; Bruker, 2015)	*k* = −10→10
*T*_min_ = 0.692, *T*_max_ = 0.746	*l* = −13→13
25254 measured reflections	

### Refinement

**Table:**

Refinement on *F*^2^	Primary atom site location: structure-invariant direct methods
Least-squares matrix: full	Secondary atom site location: difference Fourier map
*R*[*F*^2^ > 2σ(*F*^2^)] = 0.038	Hydrogen site location: mixed
*wR*(*F*^2^) = 0.103	H atoms treated by a mixture of independent and constrained refinement
*S* = 1.06	*w* = 1/[σ^2^(*F*_o_^2^) + (0.0487*P*)^2^ + 0.4447*P*] where *P* = (*F*_o_^2^ + 2*F*_c_^2^)/3
2600 reflections	(Δ/σ)_max_ < 0.001
152 parameters	Δρ_max_ = 0.38 e Å^−^^3^
0 restraints	Δρ_min_ = −0.16 e Å^−^^3^

### Special details

**Table:**

Geometry. All esds (except the esd in the dihedral angle between two l.s. planes) are estimated using the full covariance matrix. The cell esds are taken into account individually in the estimation of esds in distances, angles and torsion angles; correlations between esds in cell parameters are only used when they are defined by crystal symmetry. An approximate (isotropic) treatment of cell esds is used for estimating esds involving l.s. planes.

### Fractional atomic coordinates and isotropic or equivalent isotropic displacement parameters (Å^2^)

**Table:**

	*x*	*y*	*z*	*U*_iso_*/*U*_eq_	
N1	0.25794 (6)	−0.00837 (12)	0.18433 (9)	0.0135 (2)	
N2	0.13175 (7)	0.12269 (12)	−0.04210 (9)	0.0152 (2)	
H2	0.1258 (11)	0.1242 (19)	0.0383 (16)	0.025 (4)*	
O1	0.34355 (6)	0.09306 (11)	0.52268 (8)	0.0226 (2)	
O2	0.05883 (7)	0.20570 (12)	−0.25772 (8)	0.0254 (2)	
C1	0.19297 (8)	−0.05200 (15)	0.41627 (11)	0.0186 (2)	
H1A	0.199647	−0.175214	0.401962	0.028*	
H1B	0.139946	−0.004880	0.342575	0.028*	
H1C	0.176645	−0.033654	0.501886	0.028*	
C2	0.28862 (8)	0.03613 (14)	0.41993 (10)	0.0154 (2)	
C3	0.31963 (8)	0.04845 (13)	0.29018 (10)	0.0137 (2)	
C4	0.41926 (8)	0.12803 (15)	0.30273 (11)	0.0190 (2)	
H4A	0.471461	0.045455	0.343678	0.029*	
H4B	0.426448	0.230613	0.359472	0.029*	
H4C	0.425014	0.160061	0.213243	0.029*	
C5	0.28445 (8)	−0.02162 (14)	0.06164 (10)	0.0136 (2)	
C6	0.36956 (8)	−0.10852 (14)	0.05394 (11)	0.0155 (2)	
H6	0.413667	−0.152562	0.133672	0.019*	
C7	0.39089 (8)	−0.13174 (15)	−0.06887 (11)	0.0173 (2)	
H7	0.448663	−0.192741	−0.073564	0.021*	
C8	0.32711 (8)	−0.06509 (15)	−0.18428 (11)	0.0181 (2)	
H8	0.342227	−0.078164	−0.268210	0.022*	
C9	0.24121 (8)	0.02072 (15)	−0.17894 (11)	0.0169 (2)	
H9	0.198150	0.066045	−0.258987	0.020*	
C10	0.21796 (8)	0.04058 (13)	−0.05641 (10)	0.0137 (2)	
C11	0.05744 (8)	0.19599 (14)	−0.13980 (11)	0.0169 (2)	
C12	−0.02681 (8)	0.26801 (16)	−0.09199 (11)	0.0197 (2)	
H12A	−0.036247	0.389215	−0.117457	0.030*	
H12B	−0.011413	0.257572	0.006139	0.030*	
H12C	−0.087703	0.204217	−0.133482	0.030*	

### Atomic displacement parameters (Å^2^)

**Table:**

	*U*^11^	*U*^22^	*U*^33^	*U*^12^	*U*^13^	*U*^23^
N1	0.0143 (4)	0.0147 (4)	0.0114 (4)	0.0018 (3)	0.0033 (3)	0.0017 (3)
N2	0.0165 (4)	0.0185 (5)	0.0103 (4)	0.0018 (4)	0.0031 (3)	0.0008 (3)
O1	0.0279 (5)	0.0259 (5)	0.0124 (4)	−0.0038 (3)	0.0025 (3)	−0.0016 (3)
O2	0.0289 (5)	0.0336 (5)	0.0115 (4)	0.0096 (4)	0.0016 (3)	0.0008 (3)
C1	0.0201 (5)	0.0226 (6)	0.0155 (5)	0.0006 (4)	0.0087 (4)	0.0004 (4)
C2	0.0190 (5)	0.0144 (5)	0.0126 (5)	0.0030 (4)	0.0039 (4)	0.0013 (4)
C3	0.0143 (5)	0.0135 (5)	0.0129 (5)	0.0014 (4)	0.0032 (4)	0.0016 (4)
C4	0.0169 (5)	0.0237 (6)	0.0162 (5)	−0.0045 (4)	0.0039 (4)	−0.0019 (4)
C5	0.0150 (5)	0.0141 (5)	0.0119 (5)	−0.0030 (4)	0.0040 (4)	−0.0003 (4)
C6	0.0148 (5)	0.0174 (5)	0.0136 (5)	−0.0001 (4)	0.0026 (4)	0.0016 (4)
C7	0.0167 (5)	0.0192 (5)	0.0178 (5)	−0.0006 (4)	0.0075 (4)	−0.0014 (4)
C8	0.0226 (6)	0.0202 (6)	0.0136 (5)	−0.0028 (4)	0.0086 (4)	−0.0016 (4)
C9	0.0207 (5)	0.0179 (5)	0.0110 (5)	−0.0012 (4)	0.0027 (4)	0.0009 (4)
C10	0.0142 (5)	0.0132 (5)	0.0129 (5)	−0.0018 (4)	0.0023 (4)	−0.0007 (4)
C11	0.0178 (5)	0.0162 (5)	0.0142 (5)	0.0000 (4)	−0.0002 (4)	−0.0014 (4)
C12	0.0172 (5)	0.0232 (6)	0.0165 (5)	0.0030 (4)	0.0006 (4)	−0.0003 (4)

### Geometric parameters (Å, º)

**Table:**

N1—C3	1.2756 (14)	C4—H4C	0.9800
N1—C5	1.4147 (13)	C5—C6	1.3904 (15)
N2—H2	0.855 (15)	C5—C10	1.4053 (15)
N2—C10	1.4074 (14)	C6—H6	0.9500
N2—C11	1.3640 (14)	C6—C7	1.3888 (15)
O1—C2	1.2138 (13)	C7—H7	0.9500
O2—C11	1.2239 (14)	C7—C8	1.3831 (16)
C1—H1A	0.9800	C8—H8	0.9500
C1—H1B	0.9800	C8—C9	1.3890 (16)
C1—H1C	0.9800	C9—H9	0.9500
C1—C2	1.4954 (15)	C9—C10	1.3955 (15)
C2—C3	1.5164 (14)	C11—C12	1.5029 (16)
C3—C4	1.4990 (15)	C12—H12A	0.9800
C4—H4A	0.9800	C12—H12B	0.9800
C4—H4B	0.9800	C12—H12C	0.9800
			
C3—N1—C5	120.76 (9)	C5—C6—H6	119.6
C10—N2—H2	114.6 (10)	C7—C6—C5	120.81 (10)
C11—N2—H2	117.2 (10)	C7—C6—H6	119.6
C11—N2—C10	128.14 (9)	C6—C7—H7	120.4
H1A—C1—H1B	109.5	C8—C7—C6	119.29 (10)
H1A—C1—H1C	109.5	C8—C7—H7	120.4
H1B—C1—H1C	109.5	C7—C8—H8	119.6
C2—C1—H1A	109.5	C7—C8—C9	120.79 (10)
C2—C1—H1B	109.5	C9—C8—H8	119.6
C2—C1—H1C	109.5	C8—C9—H9	119.9
O1—C2—C1	122.86 (10)	C8—C9—C10	120.23 (10)
O1—C2—C3	118.91 (10)	C10—C9—H9	119.9
C1—C2—C3	118.20 (9)	C5—C10—N2	116.91 (9)
N1—C3—C2	116.44 (9)	C9—C10—N2	124.04 (10)
N1—C3—C4	128.13 (10)	C9—C10—C5	119.04 (10)
C4—C3—C2	115.42 (9)	N2—C11—C12	115.04 (10)
C3—C4—H4A	109.5	O2—C11—N2	123.23 (11)
C3—C4—H4B	109.5	O2—C11—C12	121.72 (10)
C3—C4—H4C	109.5	C11—C12—H12A	109.5
H4A—C4—H4B	109.5	C11—C12—H12B	109.5
H4A—C4—H4C	109.5	C11—C12—H12C	109.5
H4B—C4—H4C	109.5	H12A—C12—H12B	109.5
C6—C5—N1	121.37 (9)	H12A—C12—H12C	109.5
C6—C5—C10	119.77 (10)	H12B—C12—H12C	109.5
C10—C5—N1	118.58 (9)		
			
N1—C5—C6—C7	175.13 (10)	C6—C5—C10—N2	178.09 (9)
N1—C5—C10—N2	4.11 (14)	C6—C5—C10—C9	−2.98 (16)
N1—C5—C10—C9	−176.95 (9)	C6—C7—C8—C9	−1.56 (17)
O1—C2—C3—N1	−177.87 (10)	C7—C8—C9—C10	−0.13 (17)
O1—C2—C3—C4	1.57 (15)	C8—C9—C10—N2	−178.76 (10)
C1—C2—C3—N1	4.39 (14)	C8—C9—C10—C5	2.40 (16)
C1—C2—C3—C4	−176.18 (9)	C10—N2—C11—O2	−3.18 (18)
C3—N1—C5—C6	53.03 (15)	C10—N2—C11—C12	177.87 (10)
C3—N1—C5—C10	−133.09 (11)	C10—C5—C6—C7	1.33 (16)
C5—N1—C3—C2	−172.41 (9)	C11—N2—C10—C5	178.73 (10)
C5—N1—C3—C4	8.23 (17)	C11—N2—C10—C9	−0.15 (18)
C5—C6—C7—C8	0.95 (17)		

### Hydrogen-bond geometry (Å, º)

**Table:**

*D*—H···*A*	*D*—H	H···*A*	*D*···*A*	*D*—H···*A*
C8—H8···O1^i^	0.95	2.54	3.3286 (14)	141
C9—H9···O2	0.95	2.24	2.8523 (15)	122
C12—H12*B*···O2^ii^	0.98	2.39	3.3387 (15)	164

Symmetry codes: (i) *x*, *y*, *z*−1; (ii) *x*, −*y*+1/2, *z*+1/2.
